# A Multi‐Reassortant H3N8 Avian Influenza Virus Derived From Migratory Birds in Eastern China Exhibits Cross‐Species Transmission Potential

**DOI:** 10.1155/tbed/9889145

**Published:** 2026-08-30

**Authors:** Yunfei Guo, Zhonglong Yang, Hui Yang, Xinyu Miao, Tao Qing, Daxin Peng, Sujuan Chen

**Affiliations:** ^1^ College of Veterinary Medicine, Yangzhou University, Yangzhou 225009, Jiangsu, China, yzu.edu.cn; ^2^ Jiangsu Co-Innovation Center for the Prevention and Control of Important Animal Infectious Disease and Zoonoses, Yangzhou 225009, Jiangsu, China; ^3^ Joint Laboratory Safety of International Cooperation of Agriculture and Agricultural-Products, Yangzhou 225009, Jiangsu, China; ^4^ Jiangsu Research Centre of Engineering and Technology for Prevention and Control of Poultry Disease, Yangzhou 225009, Jiangsu, China

**Keywords:** avian influenza virus, avian-origin H3N8 subtype, cross-species transmission, phylogenetic analysis

## Abstract

Migratory birds are one of the main reservoirs and long‐distance transmission vectors of avian influenza viruses (AIVs). A migratory‐bird‐origin H3N8 subtype AIV (A/wild bird/Huadong/sy17/2024, hereafter named as WD/HDsy17/24) was isolated and identified in Jiangsu Province, Eastern China, in 2024. However, few studies have been conducted on the cross‐species transmission potential of H3N8 AIVs from migratory birds. Phylogenetic analysis indicated that the eight gene segments of WD/HDsy17/24 originated from different subtypes of AIV such as H3N8, H1, H5N1, H5N2, and H7N7 and was a multirecombinant virus. WD/HDsy17/24 virus showed a binding affinity to both SAα‐2, 3‐galactose (Gal) and SA α‐2, 6 Gal receptors, relatively weak thermal stability and pH stability. The SPF chicken pathogenicity indicated that the virus only causes mild respiratory symptoms and leads to lung hemorrhage and congestion. The mice pathogenicity indicated that the body weight of infected mice drops to the lowest value on the third day after infection, the lung‐to‐body ratio significantly increases, and inflammatory or edema lesions appear in the lungs. Moreover, this virus can infect guinea pigs through contact transmission and aerosol transmission routes from infected SPF chickens. After infection, obvious secretions can be seen in the eyes of guinea pigs. In the contact transmission group, viral shedding was detected in the nasal wash of one guinea pig at 6 days postinfection (dpi). In the aerosol transmission group, viral shedding was detected in the nasal washes of one guinea pig at both 10 and 12 dpi and in another guinea pig at 14 dpi. After 21 days, the seroconversion rate of serum antibodies in both groups is 100%. These findings underscore the need for continued surveillance of H3N8 viruses to identify circulating strains that may potentially threaten human health.

## 1. Introduction

Avian influenza (AI) is an avian infectious disease caused by AI viruses (AIVs) and is prevalent worldwide. AIVs possess numerous serotypes and pose a serious threat to the development of the poultry industry. With well‐developed water networks, distinctive geographical features, a thriving economy, and active live poultry trade, East China serves as a key migratory site and flyway for birds, which facilitates the prevalence and transmission of AIVs [1].

H3N8 subtype AIVs are mainly transmitted among wild waterfowl as natural hosts. Wild birds promote the global transmission and genetic reassortment of viruses through migration, leading to the emergence of new variants [2]. After infecting birds, H3N8 typically exhibits low pathogenicity, causing only mild clinical symptoms but can remain a zoonotic infection potential in humans. Current zoonotic studies of AIVs have focused on human infection with highly pathogenic AIVs, such as H5N1, H5N6, and H7N9 [3, 4]. Studies on the potential human risks posed by persistent adaptive mutations of low‐pathogenic subtypes remain limited. However, in April 2022, the first case of human infection with the H3N8 subtype AIV was reported in Henan Province, China [5].

Viruses continuously recombine and evolve to enhance their adaptability to their host and environment. Mutations in hemagglutinin (HA) receptor–binding sites have been shown to alter the binding properties of the virus to α‐2,3 sialic acid receptors and α‐2,6 sialic acid receptors, enabling it to infect mammals [6]. Mutations in internal genes, such as PB2, PB1, and PA, often increase the polymerase activity of viruses in mammalian cells and promote cross‐species transmission [7–9]. In recent years, genetic reassortment of AIVs in wild birds has often been accompanied by increased pathogenicity to mammals. Novel reassortant H3N8 and H9N2 subtypes of AIVs can replicate efficiently in mice and exhibit high pathogenicity and can even infect humans [10, 11]. These reports emphasize the importance of continuous surveillance of wild birds, as AIVs not only cause severe economic losses to the poultry industry but also threaten human health through interspecies transmission via intermediate hosts.

To gain a deeper understanding of the epidemic dynamics and cross‐host transmission potential of H3N8 subtype AIVs, this study conducted whole‐genome sequencing and phylogenetic analysis of a wild‐bird‐origin H3N8 AIV isolated from the Yancheng Wetland National Nature Reserve in Jiangsu Province in 2024. The pathogenicity and transmission‐related phenotypes of the virus in avian and mammalian hosts were assessed in BALB/c mice, SPF chicken, and guinea pig models. This study provides references for formulating effective monitoring and prevention strategies, as well as experimental evidence for assessing its pathogenic risk to mammals and cross‐host transmission capacity, thereby reducing its potential public health risks.

## 2. Materials and Methods

### 2.1. Ethical Statement and Facilities

This study was carried out in strict accordance with the recommendations in the “Regulations for the Administration of Laboratory Animals” issued by the Ministry of Science and Technology of the People’s Republic of China. All animal experiments were carried out according to the welfare and ethical standards of laboratory animals issued by the Jiangsu Province Laboratory Animal Management Committee and approved by the Yangzhou University Laboratory Animal Ethics Committee (Mouse Approval Number 202510034, SPF Chicken Approval Number 202510036, Guinea Pig Approval Number 202601025).

### 2.2. Isolation and Identification of Viruses

The H3N8 subtype AIV used in this study, A/wild bird/Huadong/sy17/2024 (WD/HDsy17/24), was isolated, identified, and preserved by our laboratory from the Yancheng Wetland National Nature Reserve in Jiangsu Province in 2024. Fresh fecal samples of wild birds were swabbed by field staff using sterile swabs. All samples were inoculated individually into 10‐day‐old SPF chicken embryos and incubated at 37°C for 72 h [12]. Allantoic fluids were harvested at 72 h postinoculation and stored at −80°C and tested for hemagglutination with 1% chicken red blood cells. HA and neuraminidase (NA) subtypes were determined by gene sequencing.

### 2.3. Genetic Evolution and Antigenic Site Variability Analysis

Websites and software including GISAID, ITOL, NetNGlyc‐1.0, Chiplot, PhyloSuite, Bioaider, and MegAlign were used to analyze the gene sequences of the isolate, evaluating its genetic evolution, amino acid sites, antigenic sites, and potential glycosylation site variations. PhyloSuite was used for multisequence alignment with the MAFFT algorithm, best‐fit nucleotide substitution model selection with ModelFinder under the Bayesian Information Criterion (BIC), and maximum‐likelihood (ML) tree construction with 1000 bootstrap replicates and SH‐aLRT support (1000 replicates). Trees were visualized and annotated using the ITOL and Adobe Illustrator. NetNGlyc‐1.0 was used for N‐glycosylation site prediction with a threshold of >0.5, and Chiplot was used for heatmap visualization. MegAlign was employed for nucleotide and amino acid identity calculation, and Bioaider was used for comprehensive sequence analysis. Adobe Illustrator software was used for typesetting the result figures [13–15].

### 2.4. Virus EID_50_ and TCID_50_ Determination

The virus was diluted 1:10,000 with PBS (pH 7.2) and inoculated into 10‐day‐old SPF chicken embryos at a dose of 0.2 mL/embryo. The infected allantoic fluid was aseptically collected, thoroughly mixed, aliquoted, and stored at −80°C. Fresh allantoic fluid was serially 10‐fold diluted with PBS (pH 7.2) to obtain dilutions ranging from 10^−1^ to 10^−9^. Each dilution was inoculated into five 10‐day‐old SPF chicken embryos at a volume of 0.1 mL per embryo. The infection status of chicken embryos was observed and recorded within 72 h postinoculation, and the EID_50_ of the strain was calculated using the Reed–Muench method.

CEF cells were counted and seeded into 96‐well plates at a density of 4 × 10^4^ cells per 0.1 mL per well. After 12 h of incubation, the virus was serially 10‐fold diluted in MEM and inoculated into each well at 0.1 mL per well with four replicate wells per dilution. Following viral adsorption at 37°C for 1 h, the supernatant was discarded, and the cells were washed three times with PBS. Subsequently, 0.1 mL of MEM containing 0.5 μg/mL TPCK‐trypsin was added to each well. After incubation at 37°C for 72 h, the number of positive wells was determined by the indirect immunofluorescence assay. The TCID_50_ of the strain was calculated using the Reed–Muench method.

### 2.5. Thermostability Assay

Fresh allantoic fluid from the same batch was aliquoted and incubated in a 56°C water bath. Samples were removed at 5, 15, 30, 60, 90, 120, and 180 min and immediately placed in a 4°C water bath for 5–10 min. HA titers and EID_50_ were then measured [16]. Based on whether the HA titer decreased by 2 log_2_ at 5 or 30 min at 56°C, the thermostability of the HA was classified into three types: thermolabile (2 log_2_ decrease at 5 min), thermostable (2 log_2_ decrease at 30 min), and moderately thermostable (intermediate between the two).

### 2.6. Acid Stability Assay

The virus allantoic fluid was mixed with 0.1 M citrate buffer at various pH at a ratio of 1:9 (V:V) to obtain virus suspensions at pH 4.5, 5.0, 5.5, 6.0, 6.5, and 7.0 [17] and incubated in a 37°C water bath for 15 min. HA titers were then determined by the hemagglutination assay.

### 2.7. Goose Red Blood Cell Hemagglutination Assay for Receptor‐Binding Specificity Determination

Fresh goose blood was collected and washed three times with precooled (4°C) PBS to remove components other than red blood cells. PBS was added to prepare a 10% red blood cell suspension. 1.25 U of α‐2,3 sialidase was added to each 0.1 mL of 10% goose red blood cells, followed by incubation in a 37°C water bath for 1 h. The suspension was taken out and mixed every 15 min. After 1 h, the goose red blood cells were removed from the water bath, washed three times with PBS, and adjusted to a working concentration of 1%. First, 25 μL of PBS was added to a 96‐well hemagglutination plate, and the virus was serially diluted two‐fold. The titer of the strain was determined using goose red blood cells before and after α‐2,3 sialidase treatment. A/California/04/2009/H1N1 (binding only to α‐2,6‐SA receptors) and A/Mallard/Huadong/S/2005/H5N1 (binding only to α‐2,3‐SA receptors) were set as controls. The plate was placed at 37°C for 10 min, and the hemagglutination titer results were read.

### 2.8. Solid‐Phase ELISA for Receptor‐Binding Specificity Determination

Referring to previous methods [18], briefly, 3′‑sialyllactosamine (3’SLN) and 6′‑sialyllactosamine (6’SLN) were serially diluted in sterile PBS, coated onto streptavidin ELISA plates (PBS as negative control), and incubated overnight at 4°C. After washing with PBST, plates were blocked with 5% skimmed milk at 4°C. The virus was diluted to 6 log_2_ HA units in 1% BSA‐PBS, added to the plates, and incubated overnight at 4°C. Chicken polyclonal antiserum (8 log_2_ HI units) was then added and incubated, followed by HRP‐labeled goat antichicken IgG. After washing, the TMB substrate was added, the reaction was stopped with sulfuric acid, and absorbance was measured at 450 nm. Curves were generated using GraphPad Prism 9.

### 2.9. Pathogenicity Test of the Isolate in SPF Chicken

Twelve 3‐week‐old SPF chickens were divided into an inoculated group (*n* = 6) and a negative control group (*n* = 6). The inoculated group was challenged with the virus at a dose of 10^6^ EID_50_/0.1 mL via eye and nasal drops, while the negative control group received PBS using the same route. Clinical symptoms and mortality were recorded daily for 14 days postinfection (dpi).

Oropharyngeal and cloacal swabs were collected from the inoculated group at 1, 3, 5, 7, 9, 11, and 13 dpi. Each swab was placed into 1 mL of triple‐antibiotic PBS, freeze‐thawed, and centrifuged. The supernatant was collected for viral RNA extraction using a magnetic bead–based viral genome extraction kit, followed by reverse transcription to generate cDNA. Viral shedding in oropharyngeal and cloacal swabs was quantified by absolute quantitative real‐time qPCR targeting the AIV M gene using specific primers and probes (Table 1). Viral copy numbers were calculated based on the logarithmic values derived from the standard curve.

**Table 1:** Primer and probe sequences.

Primer and probe	Sequences 5′–3′
TaqMan M–R	CTTTAKCCAYTCCATGAGAGC
TaqMan M–F	CTTCTAAGCCGAGGTCCAAAACG
M–probe	FAM–CCTTAAGCCGAGATC–MGB

*Note:* The nucleotide sequences of the TaqMan primers and probe used in this study are provided (5′ to 3′ direction). The 5′ end of the probe was labeled with FAM fluorescent dye, and the 3′ end was labeled with a minor groove binder (MGB).

At 3 dpi, three chickens from each group were randomly euthanized. Eight tissues (heart, liver, spleen, lung, brain, trachea, ileum, and nasal turbinate) were aseptically collected. After weighing, 0.1 g of each tissue was placed into a grinding tube containing 1 mL of TRIzol and homogenized. Viral loads in tissues were determined by qRT‐PCR. Portions of the lung, brain, ileum, and nasal turbinate were fixed in 4% paraformaldehyde for histopathological examination.

### 2.10. Pathogenicity Test of the Isolate in Mice

Twenty‐two 6‐week‐old female BALB/c mice were divided into an inoculated group (*n* = 11) and a negative control group (*n* = 11). In each group, five mice were designated for necropsy at 3 dpi, and the remaining six mice were used for body weight monitoring. After anesthesia with Zoletil, the inoculated group was challenged with the virus at a dose of 10^6^ EID_50_/50 μL via intranasal and ocular routes, while the negative control group received 50 μL of PBS using the same route.

At 3 dpi, five mice from each group were randomly selected and euthanized for necropsy. 0.1 g each of the heart, liver, spleen, lung, kidney, brain, nasal turbinate, trachea, and ileum was aseptically collected, placed into grinding tubes containing 1 mL of TRIzol, homogenized, and centrifuged. The supernatant was collected for viral load determination by absolute quantitative RT‐qPCR. Partial tissues of the lung, nasal turbinate, ileum, and brain were fixed in 4% paraformaldehyde for histopathological examination. Clinical signs and body weight changes were monitored daily for 14 dpi. Mice that lost more than 25% of their initial body weight were humanely euthanized for animal welfare reasons and recorded as dead.

### 2.11. Contact Transmission of the Isolate From SPF Chicken to Guinea Pigs

Six female guinea pigs weighing ~300 g were divided into a contact group (*n* = 3) and a negative control group (*n* = 3). Three 3‐week‐old SPF chickens were inoculated with the isolate via eye and nasal drops at a dose of 10^6^ EID_50_/0.1 mL. PBS was used to inoculate the negative control group and contact group guinea pigs. Guinea pigs were anesthetized with diluted Zoletil and inoculated intranasally [19].

At 24 h after SPF chicken inoculation, guinea pigs serving as contact exposure animals and inoculated SPF chicken were placed into a single shared isolator to undergo cohabitation contact challenge. At days 2, 4, 6, 8, 10, 12, and 14 postcontact, the nasal cavity of each cohoused guinea pig was flushed with triple‐antibiotic PBS to collect 1 mL of nasal wash per guinea pig. RNA was extracted and reverse‐transcribed, and viral shedding was detected by RT‐qPCR. Twenty‐one days postcontact, fresh blood was collected from cohoused guinea pigs via cardiac puncture, serum was separated, and the serum hemagglutination inhibition (HI) titer was detected by the HI assay.

### 2.12. Aerosol Transmission of the Isolate From SPF Chicken to Guinea Pigs

Six female guinea pigs weighing ~300 g were divided into an aerosol transmission group (*n* = 3) and a negative control group (*n* = 3). Three 3‐week‐old SPF chickens were inoculated with the H3 isolate via eye and nasal drops at a dose of 10^6^ EID_50_/0.1 mL. PBS was used to inoculate the negative control group and the aerosol transmission group guinea pigs. Guinea pigs were anesthetized with diluted Zoletil and inoculated intranasally.

Twenty‐four hours after chicken inoculation, the aerosol transmission group guinea pigs and the inoculated SPF chicken were housed in separate isolators placed 50 cm apart. The guinea pig isolator was positioned downwind of the airflow from the chicken isolator, ensuring unidirectional airflow from the chicken isolator toward the guinea pig isolator to prevent cross‐contamination between the two groups. One milliliter of nasal wash was collected from each guinea pig at 2, 4, 6, 8, 10, 12, and 14 dpi, and the virus shedding titer and serum HI titer were detected using the same methods.

## 3. Results

### 3.1. Genetic Evolution and Antigenic Site Variability Analysis of the Isolate

Phylogenetic trees were constructed using the ML method based on sequences of all eight gene segments of H3 subtype AIVs from different hosts downloaded from the GISAID database. Both the HA and NA genes of the WD/HDsy17/24 isolate clustered with wild‐bird‐origin H3N8 viruses. Meanwhile, homology analysis of the HA and NA genes between the WD/HDsy17/24 strain with downloaded reference sequences was performed via heatmap visualization. The HA gene of the isolate formed a distinct clade separate from poultry, swine‐origin, and human‐origin H3 subtype AIVs (Figure 1A). Heatmap results indicated that the HA gene sequences exhibited large differences in homology with H3 subtype isolates from different hosts (Figure 1B,C). The NA gene exhibited close genetic distance to several human‐origin H3N8 isolates from recent years (Figure 2A). Compared with the HA gene, the NA gene of the isolate showed higher nucleotide and amino acid homology with wild‐bird‐origin and human‐origin H3N8 subtype AIVs from the past 15 years (Figure 2B,C). The M, NS, NP, PB2, and PA genes of the isolate all belonged to the Eurasian lineage, whereas the PB1 gene belonged to the North American lineage (Figure 3). The M and NS genes showed close genetic distance to H9N2 subtype AIVs and the human‐origin H10N3 strain 0428. Gene origin analysis was performed by comparing with viruses in the GISAID database that had the highest homology with each segment of the corresponding virus. The eight gene segments shared high homology (>98%) with those of H3N8, H5N1, H5N2, H3N2, H1, and H7N7 subtype AIVs in the GISAID database (Table 2).

Figure 1Phylogenetic and homology analysis of the HA gene of the H3N8 isolate WD/HDsy17/24. (A) Phylogenetic tree of the HA gene of H3 subtype avian influenza viruses. The tree was constructed using the maximum‐likelihood (ML) method with 1000 bootstrap replicates. Bootstrap values are shown at the branch nodes. The HA gene of WD/HDsy17/24 is highlighted in red. (B) Heatmap showing nucleotide sequence identity of the HA gene between WD/HDsy17/24 and other H3 subtype isolates. (C) Heatmap showing amino acid sequence identity of the HA gene between WD/HDsy17/24 and other H3 subtype isolates. Warmer colors represent higher sequence identity.

**Figure:**
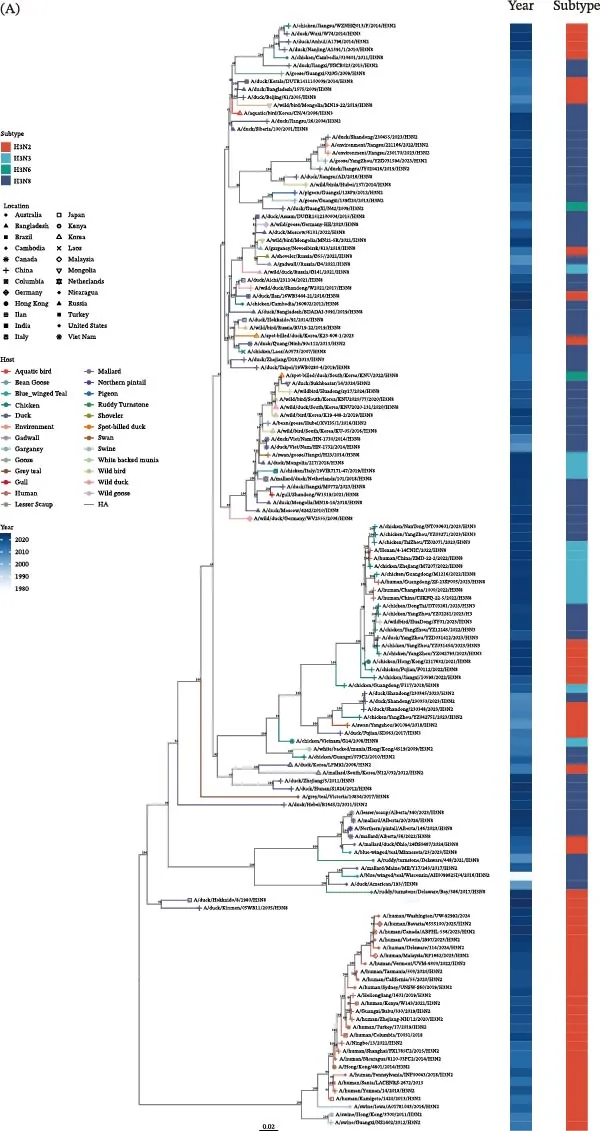

**Figure:**
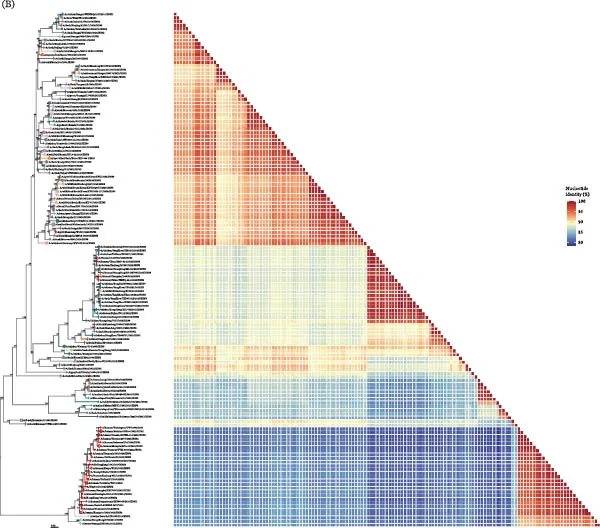

**Figure:**
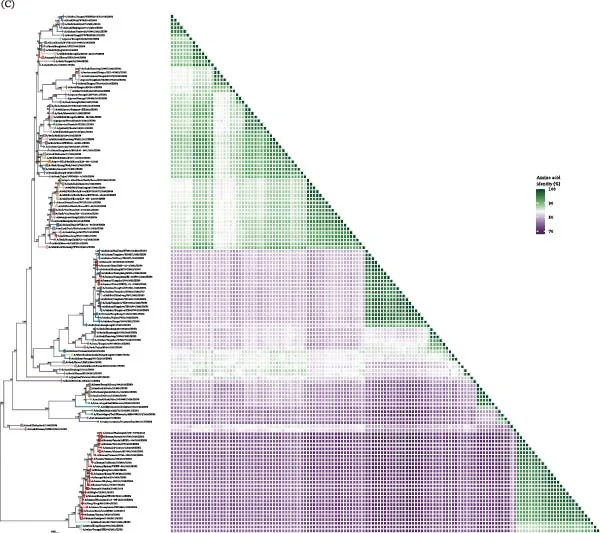

Figure 2Phylogenetic and homology analysis of the NA gene of the H3N8 isolate WD/HDsy17/24. (A) Phylogenetic tree of the NA gene of H3N8 subtype avian influenza viruses. The tree was constructed using the maximum‐likelihood (ML) method with 1000 bootstrap replicates. Bootstrap values are shown at the branch nodes. The NA gene of WD/HDsy17/24 is highlighted in red. (B) Heatmap showing nucleotide sequence identity of the NA gene between WD/HDsy17/24 and other H3 isolates. (C) Heatmap showing amino acid sequence identity of the NA gene between WD/HDsy17/24 and other H3 isolates. Warmer colors represent higher sequence identity.

**Figure:**
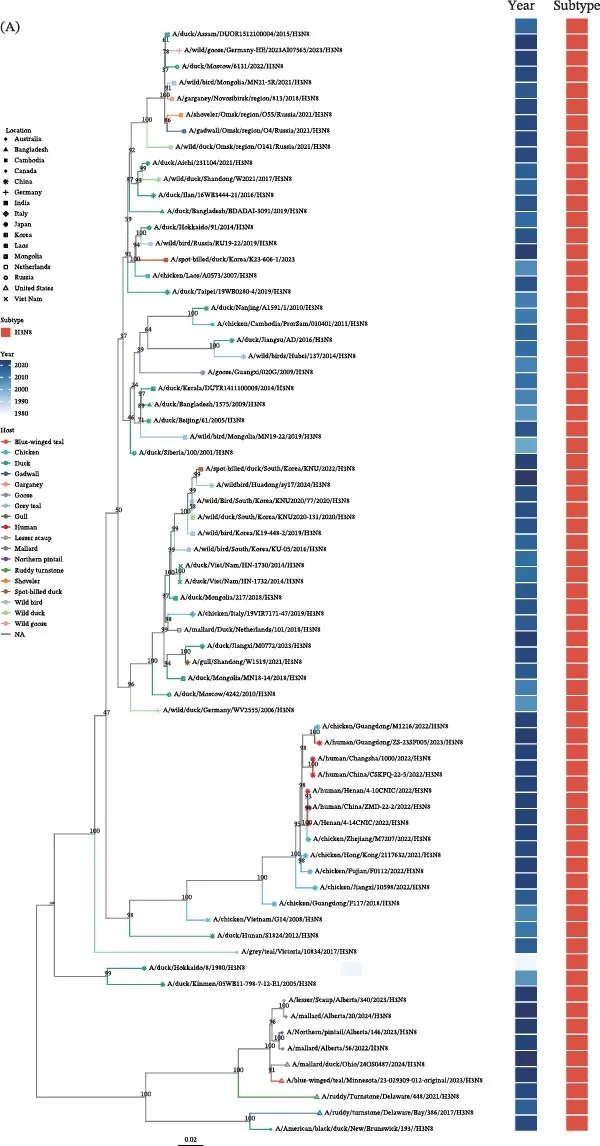

**Figure:**
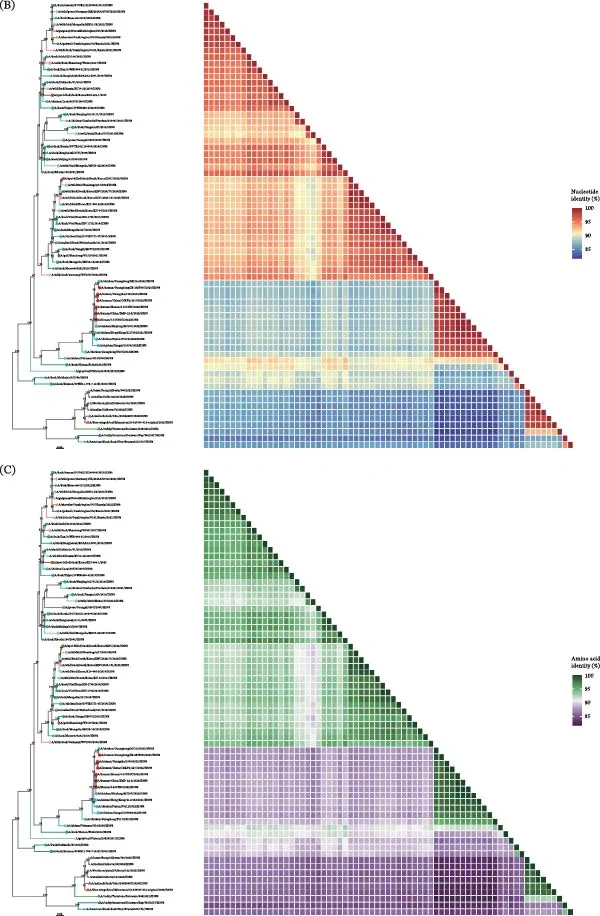

Figure 3Phylogenetic analysis of the six internal genes of the H3N8 isolate WD/HDsy17/24. (A) M gene. (B) NS gene. (C) NP gene. (D) PA gene. (E) PB1 gene. (F) PB2 gene. Phylogenetic trees of the M, NS, NP, PB2, PB1, and PA genes were constructed using the maximum‐likelihood (ML) method with 1000 bootstrap replicates. Bootstrap values are shown at the branch nodes. The internal genes of WD/HDsy17/24 are highlighted in red. The M, NS, NP, PB2, and PA genes all belonged to the Eurasian lineage (blue shading), whereas the PB1 gene belonged to the North American lineage (red shading).

**Figure:**
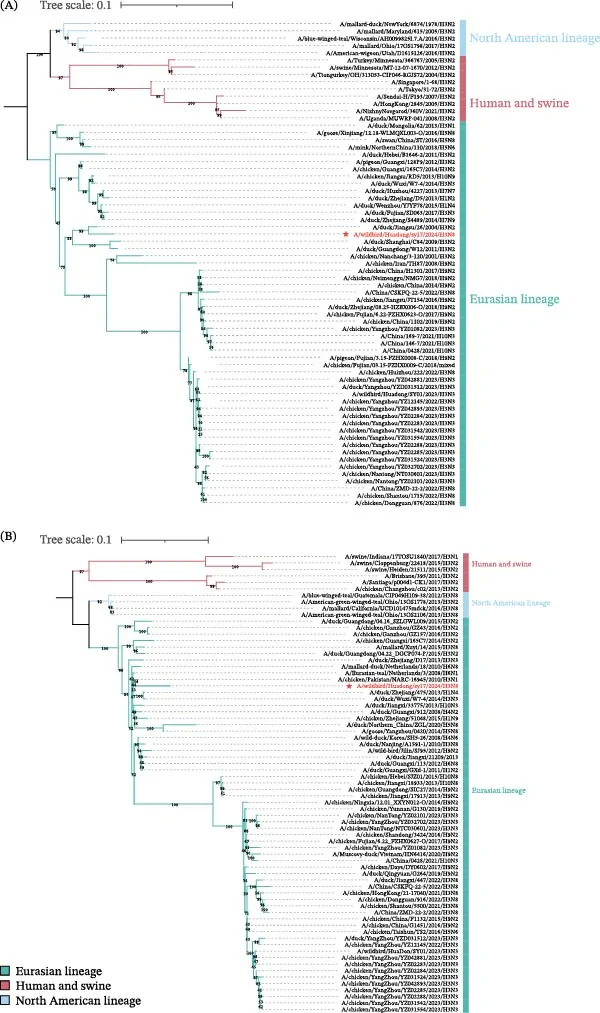

**Figure:**
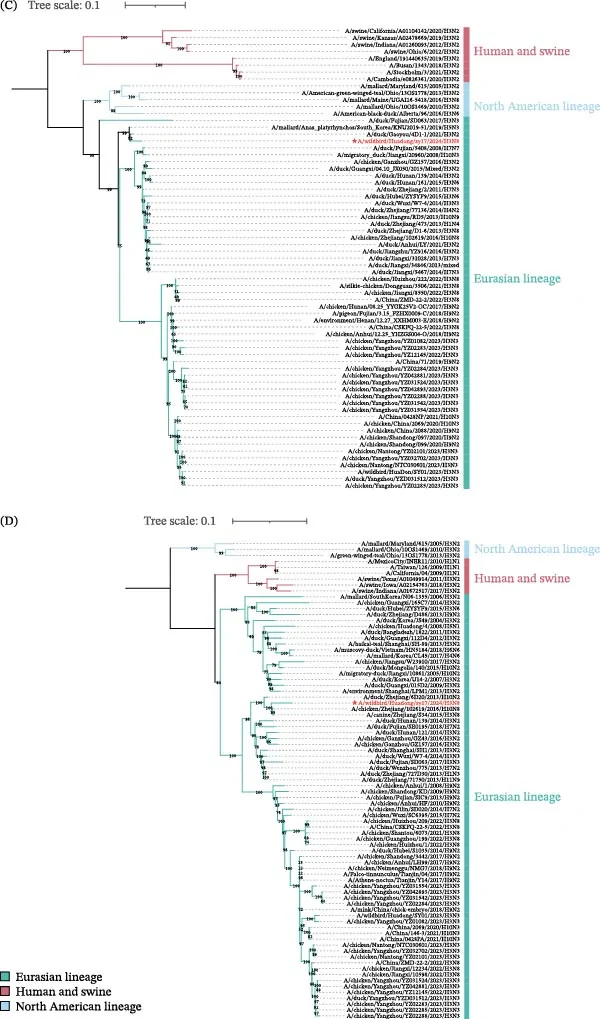

**Figure:**
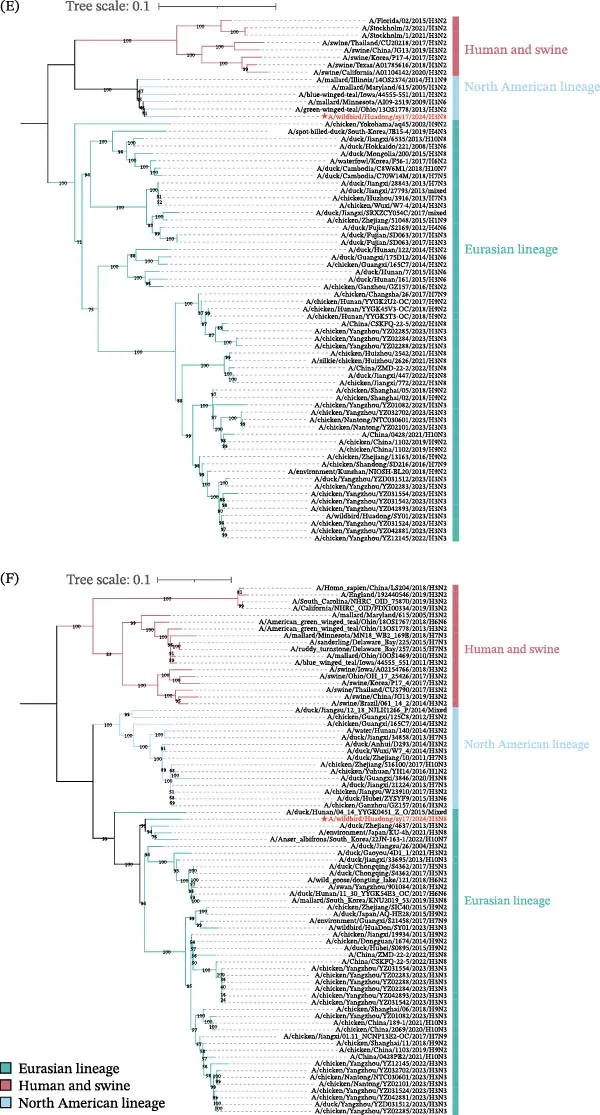

**Table 2:** Homology analysis of each gene segment from H3N8.

Gene	The virus with the highest sequence homology	Homology rate (%)	Accession number
HA	A/Spot‐billed/duck/South/Korea KNU2022‐20/2022(H3N8)	99	EPI_ISL3268126
NA	A/Garganey/Yakutia/K‐74/2023(H3N8)	99	EPI_ISL3377647
PB2	A/duck/Korea/D051/2025(H5N1)	99	EPI_ISL4393698
PB1	A/northern shoveler/Alaska/17‐004479‐3/2016 (H5N2)	98	EPI_ISL_397839
PA	A/pigeon/China/286P45/2017 (H3N2)	99	EPI_ISL19243994
NP	A/environment/Bangladesh/17‐E‐106/2023 (H1)	98	EPI_ISL3116420
M	A/duck/Tainan/16WB3443‐16‐20/2016(H7N7)	99	EPI_ISL_19226396
NS	A/Teal/Khasanskiy district/2061/2025(H3N8)	99	EPI_ISL5090291

*Note:* A homology search was performed using the BLAST program implemented in GenBank, targeting sequences highly homologous to the assembled gene sequences of isolate WD/HDsy17/24.

Sequence determination results showed that the full‐length ORF of the HA gene of the isolate was 1701 bp, encoding 567 amino acids. The HA1 region encoded 344 amino acids, and the HA2 region encoded 221 amino acids, without nucleotide insertions or deletions. The HA protein cleavage site was ^340^PEKQTR↓GLF^348^, which is consistent with the molecular characteristics of low‐pathogenic AIVs. The full‐length ORF of the NA gene was 1413 bp, encoding 471 amino acids.

Analysis of potential glycosylation sites showed that, in addition to five conserved N‐glycosylation sites at positions 38, 54, 181, 301, and 499 on the HA peptide chain, a new glycosylation site (NST) at position 24 was added. This site was also present in the human‐origin reference strain A/Hong Kong/1/1968 (H3N2) and the swine‐origin reference strain A/swine/Guangdong/423/2006 (H3N2) (Table 3). Potential glycosylation sites at positions 46, 54, 84, 144, 293, and 398 were present in the NA sequence of the isolate, and no deletion occurred in the stalk region of the NA protein.

**Table 3:** Analysis of potential N‐glycosylation sites in the HA protein of H3 AIV.

Strains	Potential glycosylation sites of HA proteins					
	24	38	54	181	301	499
	NST	NVT	NGT	NSS	NGT	NGS
WD/HDsy17/24	+	+	+	+	+	+
A/chicken/Jiangsu/W23910/2017/H3N2	−	+	+	+	+	+
A/HongKong/1/1968/H3N2	+	+	+	+	+	+
A/swine/Guangdong/423/2006/H3N2	+	+	+	+	+	−

*Note:* The NetNGlyc‐1.0 web server was used to predict potential N‐glycosylation sites of the isolate, and the results were further compared with those of reference strains of avian, swine, and human origins. + indicates the presence of a potential glycosylation site; − indicates its absence. The amino acid motif at each position is shown in parentheses below the position number.

Adaptive amino acid site analysis of the isolate is shown in Table 4. The HA‐T160A mutation can increase the virus’s binding ability to human‐type receptors, and the HA‐N224K mutation can alter the virus’s receptor–binding properties [20]. The triple mutations N30D, I43M, and T215A in the M1 protein and the double mutations P64S and L69P in the M2 protein are related to host adaptability and have been shown to significantly enhance the pathogenicity of the virus in mice [21–23]. The NS1 protein possesses key amino acid mutations, including P42S, L103F, I106M, and N209D that can improve the virus’s replication ability in mammals. The PDZ domain sequence at amino acid positions 227–230 at the C‐terminus of the NS1 protein is ESEV, which can enhance the pathogenicity of the virus in mouse models [24]. The NP‐H289Y mutation is related to adaptive mutations in mice. The PB1‐V171M, PB1‐R198K, and PB1‐H436Y mutations can increase the adaptability of the strain to mice, and the PB1‐L473V mutation can enhance the virus’s transmission ability in mammalian cells [25, 26]. PB2 has I495V and R477G variations that can increase the virulence and replication ability of the virus in mice [27]. PA‐A100V, PA‐R356K, and PA‐N409S mutations can increase the polymerase activity of the virus. The PA‐L295P mutation can enhance adaptability to mice. The PA‐N383D mutation can enhance pathogenicity to ducks. The PA‐F666L mutation can improve the virus’s ability to replicate in mammals [23].

**Table 4:** Analysis of molecular characteristics of amino acids.

Protein	Amino acid position/motif	Phenotypic consequences
PB2	I495V, R477G	Increased virulence and replication in mice
PB1	V171M, R198K, H436Y	Increased virulence in mice
	L473V	Increased transmission in mammalian cells
HA	T160A	Increased virus binding to human‐type receptor
	N224K	Change the receptor‐binding properties
M1	N30D, I43M, T215A	Increased virulence in mice
M2	P64S, L69P	Increased virulence in mice
NS1	P42S, L103F, I106M, N209D	Increased virulence in mice
PA	A100V, R356K, N409S	Increased virulence in mice and increased polymerase activity
	L295P	Increased virulence in mice
	N383D	Increased virulence in duck
	F666L	Increased virus replication in mammals
NP	H289Y	Adaptive mutations in mice

*Note:* The MegAlign software was employed to analyze the gene sequences of the test strain to evaluate the variation profiles of its amino acid sites and antigenic sites. The phenotypic consequences of each mutation are described.

### 3.2. Determination of EID_50_ and TCID_50_ of the Isolate

The EID_50_ of the H3N8 isolate was calculated to be 10^7^·^67^ EID_50_/0.1 mL using the Reed–Muench method. The TCID_50_ of the H3N8 isolate was calculated to be 10^5^·^5^ TCID_50_/mL on CEF using the Reed–Muench method.

### 3.3. Thermostability Assay

**Figure 4:**
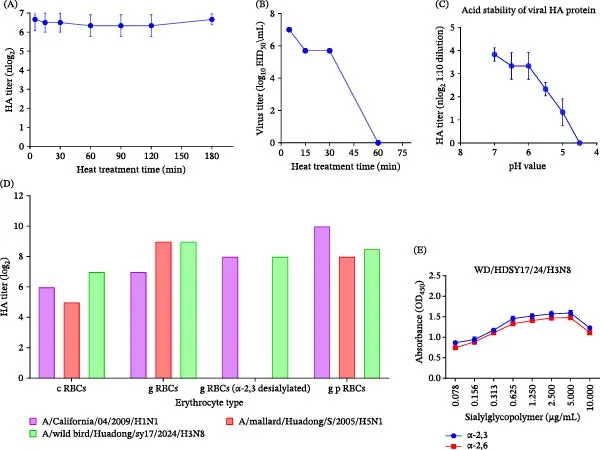
Thermostability, acid stability, and receptor‐binding specificity of the H3N8 isolate WD/HDsy17/24. (A) Hemagglutination (HA) activity of the isolate after incubation at 56°C for the indicated times. The virus retained full HA activity after 180 min of incubation. (B) Infectivity (EID_50_) of the isolate after incubation at 56°C for the indicated times. Infectivity was completely lost after 60 min of incubation. (C) Acid stability of the hemagglutinin. Virus allantoic fluid was diluted 1:9 (v/v) with buffers at the indicated pH values and incubated at 37°C for 15 min, and HA titers were measured. The HA titer dropped to 0 at pH 4.5. (D) Hemagglutination activity of the isolate with different erythrocyte types: chicken RBCs (cRBCs), goose RBCs (gRBCs), α‐2,3‐desialylated goose RBCs, and guinea pig RBCs (gpRBCs). The reference strains A/California/04/2009 (H1N1, human‐like α‐2,6 preference) and A/Mallard/Huadong/S/2005 (H5N1, avian‐like α‐2,3 preference) were included as controls. HA titers are expressed as log_2_ values. (E) Solid‐phase ELISA for receptor‐binding specificity. The isolate bound to both α‐2,3‐linked sialic acid (3′SLN) and α‐2,6‐linked sialic acid (6′SLN) receptors, indicating dual receptor‐binding specificity. Data are presented as mean ± SD of three independent experiments.

The isolate WD/HDsy17/24/H3N8 retained full HA activity after 180 min of incubation (Figure 4A) but completely lost infectivity after 60 min of incubation (EID_50_ was undetectable) at 56°C (Figure 4B). These results indicate that at 56°C, the HA of the isolate exhibits high thermostability, but the overall infectivity of the virus has low thermostability.

### 3.4. Acid Stability Assay

The virus allantoic fluid was diluted 1:9 (V/V) and incubated with buffers at pH 4.5, 5.0, 5.5, 6.0, 6.5, and 7.0, and the HA titers were measured. Three independent replicate experiments showed that the hemagglutination activity of the isolate was significantly reduced at pH ≤ 5.0, with the HA titer dropping to 0 at pH 4.5, indicating a complete loss of hemagglutination activity (Figure 4C). In the pH range of 6.0–7.0, the HA titer remained stable, indicating that this H3 isolate has poor acid stability and begins to inactivate under weakly acidic conditions.

### 3.5. Goose Red Blood Cell Adsorption Assay and Solid‐Phase ELISA for Receptor‐Binding Specificity Determination

The receptor‐binding specificity of the isolate was determined using the goose red blood cell adsorption assay combined with solid‐phase ELISA technology. The hemagglutination titer of the strain to treated goose red blood cells decreased from 9 to 8 log_2_, indicating that it has the ability to bind both α‐2,3 and α‐2,6 sialic acid receptors (Figure 4D). Solid‐phase ELISA results further confirmed that the strain can specifically recognize both α‐2,3 and α‐2,6‐SA receptors, exhibiting dual receptor–binding characteristics (Figure 4E).

### 3.6. Pathogenicity of the H3N8 Subtype AIV Isolate in SPF Chicken

**Figure 5:**
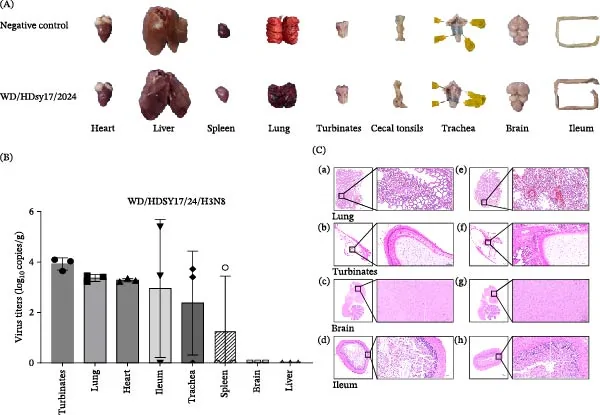
Pathogenicity of the H3N8 isolate WD/HDsy17/24 in SPF chicken. (A) Gross pathological changes in SPF chicken at 3 dpi. Lungs showed hemorrhage and congestion. Hemorrhage was also observed in the larynx and cecal tonsils. (B) Viral loads in various organs of SPF chicken at 3 dpi. Viral RNA was detected by qRT‐PCR targeting the influenza A virus M gene. Viral loads were higher in the trachea, nasal turbinates, and ileum than in other organs. Data are shown as mean ± SD of three chickens. (C) Histopathological analysis of multiple tissues in SPF chicken at 3 dpi. (a–d): Negative control group: (a) lung, (b) nasal turbinate, (c) brain, and (d) ileum. (e–h): Infection group: (e) lung, (f) nasal turbinate, (g) brain, and (h) ileum. HE staining revealed hemorrhage and congestion in local peribronchial and interstitial areas of the lung in infected chicken, with marked dilation and congestion of respiratory capillaries. No obvious histopathological lesions were observed in the nasal turbinate, brain, or ileum of either group. Scale bars: 100 μm.

All SPF chickens inoculated intranasally and ocularly with the WD/HDsy17/24 strain at a dose of 10^6^ EID_50_/0.1 mL showed no obvious clinical symptoms. At 3 dpi, three SPF chickens were randomly euthanized and necropsied. Gross pathological lesions including pulmonary congestion and hemorrhage, as well as hemorrhages in the larynx and cecal tonsils, were observed (Figure 5A). Virus titers were detected in the heart, liver, spleen, lung, brain, nasal turbinates, trachea, and ileum from the three euthanized chickens. The viral loads in the trachea, nasal turbinate, and ileum were slightly higher than those in other organs, with the mean viral titers of 3.56 ± 0.23, 3.92 ± 0.24, and 4.43 ± 1.38 log_10_ copies/g, respectively (Figure 5B).

Localized hemorrhage and congestion occurred in the peribronchial and interstitial tissues of the lungs in the infected SPF chicken, along with marked dilation and hyperemia of pulmonary capillaries (Figure 5C, e). No remarkable pathological lesions were observed in the ileum, brain, and nasal turbinates (Figure 5C, f–h). Additionally, no histopathological alterations were detected in any organs of the chicken in the control group (Figure 5C, a–d).

Throat and cloacal swabs were collected at 1, 3, 5, 7, 9, 11, and 13 dpi. Detectable viral shedding was observed in both oropharyngeal and cloacal swabs of infected chickens (Table 5). Oropharyngeal shedding was mainly detected in the early stage of infection, peaking at 1 dpi, after which the viral load gradually decreased. In contrast, cloacal shedding showed an increasing trend within 13 dpi.

**Table 5:** Viral loads of H3 subtype avian influenza viruses (AIVs) in chicken oropharyngeal and cloacal swabs (*n* = 3).

Group	Sampling time						
	dpi 1	dpi 3	dpi 5	dpi 7	dpi 9	dpi 11	dpi 13
Throat swab specimen	3.55 ± 0.46 (3/3)	3.47 ± 0.25 (3/3)	3.58 ± 0.29 (3/3)	3.44 ± 0.04 (3/3)	3.50 ± 0.11 (3/3)	3.47 ± 0.16 (3/3)	3.39 ± 0.12 (3/3)
Cloacal swab specimen	3.55 ± 0.18 (3/3)	3.64 ± 0.12 (3/3)	3.72 ± 0.18 (3/3)	3.79 ± 0.11 (3/3)	3.72 ± 0.11 (3/3)	3.74 ± 0.14 (3/3)	3.71 ± 0.02 (3/3)
Negative control group	0	0	0	0	0	0	0

*Note:* SPF chickens were inoculated with the H3N8 isolate WD/HDsy17/24 at a dose of 10^6^ EID_50_/0.1 mL (*n* = 3). Throat and cloacal swabs were collected at the indicated dpi. Viral loads (log_10_ copies/g) were determined by qRT‐PCR targeting the influenza A virus M gene. Data are shown as mean ± SD for positive samples, with the number of positive chicken/total chicken indicated in parentheses. The negative control group (PBS‐treated) showed no viral shedding.

### 3.7. Pathogenicity of the H3N8 Subtype AIV Isolate in Mice

After infecting mice with the WD/HDsy17/24 strain at a dose of 10^6^ EID_50_/mL via the intranasal route, no deaths occurred in the inoculated group within 14 days (Figure 6A), and no obvious clinical symptoms were observed. Body weight significantly decreased at 1–3 dpi (Figure 6B). Compared with the PBS control group, the lung‐to‐body weight ratio of five randomly necropsied mice in the inoculated group at 3 dpi was significantly increased (Figure 6C). Pulmonary congestion and hemorrhage as well as tracheal hemorrhage were observed, while no significant lesions were found in other organs (Figure 6D).

Figure 6Pathogenicity of the H3N8 isolate WD/HDsy17/24 in BALB/c mice. (A) Survival curve of mice after infection with the isolate. Mice were inoculated intranasally with 10^6^ EID_50_/50 μL of the virus (*n* = 6 per group). Survival rate was monitored daily for 14 dpi. No mortality was observed. (B) Body weight changes of mice infected with the isolate. Mice were inoculated intranasally with 10^6^ EID_50_/50 μL of the virus (*n* = 6 per group). Body weight was monitored daily for 14 dpi. Data are presented as mean ± SD. (C) Lung‐to‐body weight ratio of mice at 3 dpi (*n* = 5 per group). Body weight and lung weight were measured from five randomly euthanized mice at 3 dpi, and the lung‐to‐body weight ratio was calculated. An independent samples *t*‐test was used to analyze the difference in lung‐to‐body weight ratio between the PBS control group and the challenge group. The lung‐to‐body weight ratio was significantly increased in the challenge group compared to the PBS control group.  ^∗∗∗^, *p* < 0.001. (D) Gross anatomical changes in mice at 3 dpi. Representative images showing lung congestion and hemorrhage, as well as tracheal hemorrhage, in the challenge group compared to the PBS control group. (E) Histopathological analysis of multiple tissues in mice at 3 dpi. (a–d): Negative control group: (a) lung, (b) nasal turbinate, (c) brain, and (d) ileum. (e–h): Infection group: (e) lung, (f) nasal turbinate, (g) brain, and (h) ileum. HE staining showed widened alveolar septa with extensive infiltration of macrophages and lymphocytes, and bronchiolar lumina filled with inflammatory exudates. No obvious histopathological lesions were observed in the nasal turbinate, brain, or ileum of either group. Scale bars: 100 μm. (F) Viral loads in various organs of mice at 3 dpi. Viral RNA was detected by qRT‐PCR targeting the influenza A virus M gene. Relatively higher viral titers were detected in the respiratory system, including the lung and trachea. Data are shown as mean ± SD of five mice.

**Figure:**
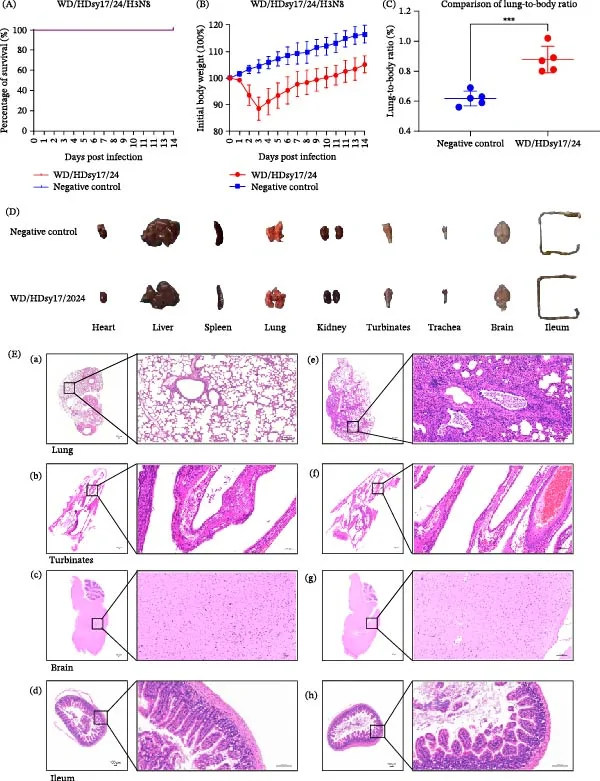

**Figure:**
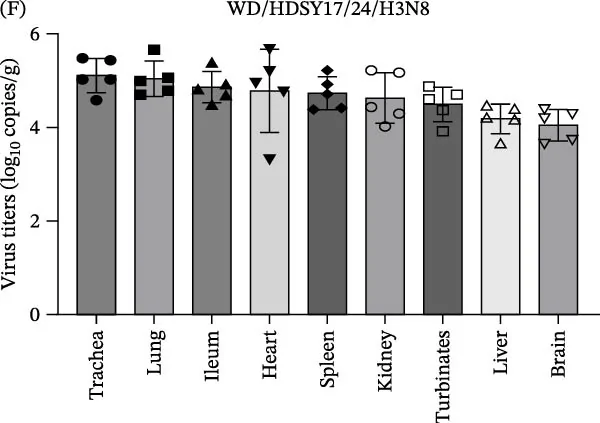

HE staining results showed that compared with the PBS control group (Figure 6E, a–d), mice infected with the isolate exhibited widened alveolar septa with extensive infiltration of macrophages and lymphocytes. Increased numbers of macrophages were observed within the alveolar spaces, with scattered erythrocytes in a small proportion of alveoli. Perivascular areas showed dense infiltration of lymphocytes and a few neutrophils. Local pulmonary vascular dilation and congestion were observed. Bronchiolar lumina were filled with inflammatory exudates, consisting mainly of necrotic and sloughed mucosal epithelial cells, along with exuded neutrophils, lymphocytes, and a small amount of blood (Figure 6E, e); no significant pathological changes were detected in the ileum, brain, or nasal turbinates (Figure 6E, f–h).

Viral titration analysis of mouse organs showed that in the five randomly necropsied mice at 3 dpi, viral replication was detected in all organs (Figure 6F).

### 3.8. Transmissibility Study of the H3N8 Subtype AIV Isolate From SPF Chicken to Guinea Pigs

**Figure 7:**
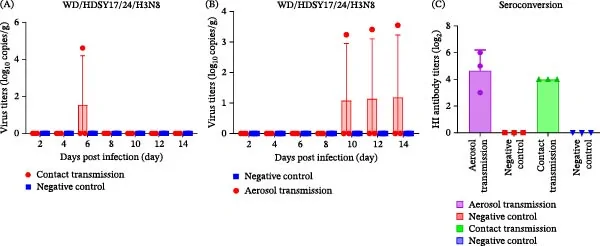
Transmissibility of the H3N8 isolate WD/HDsy17/24 from SPF chicken to guinea pigs. (A) Viral shedding in nasal washes of guinea pigs after contact transmission. Guinea pigs were cohoused with infected SPF chicken, and nasal washes were collected at the indicated days postcontact. Viral RNA was detected by qRT‐PCR. Viral shedding was observed in one guinea pig at day 6 (4.61 log_10_ copies/g). (B) Viral shedding in nasal washes of guinea pigs after aerosol transmission. Guinea pigs were housed 50 cm apart from infected SPF chicken. Viral RNA was detected at the same time points. Viral shedding was detected in the nasal wash of one guinea pig at 10 and 12 dpi and in another guinea pig at 14 dpi (3.24, 3.41, and 3.55 log_10_ copies/mL), respectively. (C) Serum hemagglutination inhibition (HI) titers of guinea pigs at 21 days postexposure. HI titers ranged from 4 to 5 log_2_. According to WOAH standards, a serum titer of ≥4 log_2_ is considered positive. All guinea pigs in both transmission groups were seropositive (100%, *n* = 3 per group). Data are presented as mean ± SD. In the figure, red bars represent the negative control for the aerosol transmission group, and blue bars represent the negative control for the contact transmission group.

Nasal lavage fluid was collected at days 2, 4, 6, 8, 10, 12, and 14 postcontact or postinfection for viral shedding detection. In the contact transmission group (Figure 7A), viral shedding was detected in the nasal wash of only one guinea pig at 6 days postcontact, with a viral titer of 4.61 log_10_ copies/mL. In the aerosol transmission group (Figure 7B), viral shedding was detected in the nasal wash of one guinea pig at 10 and 12 dpi and in another guinea pig at 14 dpi, with viral loads of 3.24, 3.41, and 3.55 log_10_ copies/mL, respectively. Serum specimens were collected from all guinea pigs at day 21 postexposure. HI titers ranged from 4 to 5 log_2_ (Figure 7C). All guinea pigs in both transmission groups were seropositive, with a seropositivity rate of 100%.

## 4. Discussion

As a natural reservoir, wild birds facilitate transregional and cross‐national transmission [28]. H3N8 AIVs, a dominant subtype in wild birds, have a broad host range, infecting poultry [29] and crossing species barriers to infect horses, dogs, pigs, and humans [30–33]. Therefore, long‐term surveillance in wild bird migration areas is urgently needed.

Recent reports have documented novel reassortant H3N8 variants isolated a reassortant H3N8 from a wild bird in Jiangxi has become widespread in Chinese chicken flocks, forming a triple‐reassortant genotype [5, 10]. The isolate WD/HDsy17/24 also displayed multi‐reassortant characteristics, with eight gene segments derived from reassortment of H1, H3N8, H5N1, H5N2, and H7N7 influenza viruses. This is consistent with a broader analysis showing that wild‐bird‐origin H3N8 in China evolved into at least 126 genotypes from 2009 to 2022, with genotype G25 (reassorted with H9N2) capable of infecting chicken and crossing to humans [34]. The close genetic relationship of our isolate’s M and NS genes to H9N2 AIVs suggests enhanced host adaptability via reassortment.

Mammalian adaptation characteristics of H3N8 viruses are often associated with specific amino acid substitutions. The HA‐T160A and N224K mutations, both present in our isolate, have been shown to enhance binding to human‐type α‐2,6–linked sialic acid receptors and to alter receptor‐binding specificity [18]. These mutations endow the virus with dual receptor–binding capacity (α‐2,3 and α‐2,6), a property also reported in a chicken‐origin H3N8 strain from Hong Kong that achieved human infection [35]. However, receptor‐binding assays reflect in vitro affinity and may not fully recapitulate the complex receptor environment and dynamic interactions that occur during viral entry in vivo. Furthermore, the PB2‐I495V and R477G mutations, detected in our strain, have been linked to increased viral replication and virulence in mice [26]; the PB1‐L473V mutation improves transmission efficiency in mammalian cells, and the PA‐F666L mutation elevates viral replication levels in mammals [36]. The combination of these adaptive mutations in our isolate suggests considerable cross‐species transmission potential, aligning with the observation by Liang et al. [31] that wild‐bird‐origin H3N8 viruses can replicate in mammals without prior adaptation.

The thermal stability of the HA protein is an important determinant of influenza virus environmental persistence. The isolate retained full HA activity after 180 min at 56°C, whereas its infectivity was lost within 60 min. This dissociation between HA thermostability and overall viral infectivity has also been observed by Mei et al. [16] in chicken‐origin H3N8 strains, suggesting that other virion components (e.g., envelope integrity or ribonucleoprotein complexes) are more heat‐labile. Acid stability is another critical factor for viral survival and transmission [37]. The isolate exhibited poor acid stability, with complete loss of hemagglutination activity at pH 4.5, a feature consistent with other H3 subtype AIVs.

In SPF chicken, the virus caused only mild respiratory symptoms, but viral replication was detected in multiple organs, including the lungs, liver, and spleen, accompanied by pulmonary hemorrhage and congestion. This low‐pathogenicity phenotype is typical for H3N8 in birds, as reported by Sit et al. [35] and Lin et al. [38]. Oropharyngeal virus shedding peaked at 1 dpi, after which the viral load gradually decreased, whereas cloacal shedding showed a gradual increase in viral load within 13 dpi. These patterns are consistent with the theoretical findings reported in previously published literature, whereby low‐pathogenic AIVs primarily replicate and shed within the first week following respiratory tract infection [39] and that the digestive tract can serve as a long‐term shedding site [40]. Individual variations in virus shedding (e.g., one chicken exhibited a very high viral load in the ileum, reaching 5.41 log_10_ copies/g, and its viral loads in other organs were also higher than those in the other two chickens) likely reflect differences in viral tropism and host immune responses [41].

In BALB/c mice, the isolate induced transient body weight loss (1–3 dpi) and a significantly increased lung‐to‐body weight ratio but no mortality. Viral replication was detected in all tested organs, with higher titers in the respiratory tract, and histopathological examination revealed alveolar septal widening, inflammatory cell infiltration, and bronchiolar exudates. These findings are consistent with previous studies on wild‐bird‐origin H3N8 viruses in mice [42]. Notably, the lesions observed here were more severe than those induced by a chicken‐origin H3N8 strain reported by Mei et al. [16], suggesting that this wild‐bird‐origin isolate may possess higher intrinsic pathogenicity in mammals. Finally, the virus was transmitted to guinea pigs via both direct contact and aerosol routes, as evidenced by viral RNA in nasal washes and seroconversion (HI titers of 4–5 log_2_). The demonstration of aerosol transmission in guinea pigs is concerning, although the relatively low viral titers in nasal lavage fluid (peak 4.61 log_10_ copies/g in contact and 3.55 log_10_ copies/g in aerosol groups) suggest limited transmission efficiency.

In summary, through analysis of the genetic evolutionary characteristics and pathogenicity of a wild‐bird‐origin H3N8 AIV from eastern China in 2024, this study confirmed that the strain carries multiple mammalian–adaptive mutations and exhibits dual receptor–binding capacity and cross‐species transmission potential. The findings provide important experimental data for the early warning, surveillance, risk assessment, and control strategy development of H3N8 AIVs and are of great significance for mitigating their potential public health risks.

## Author Contributions

**Sujuan Chen**: writing – review and editing, writing – original draft, software, methodology, investigation, funding acquisition, data curation, conceptualization. **Daxin Peng**: writing – review and editing, writing – original draft, investigation, funding acquisition. **Tao Qing**: funding acquisition, conceptualization. **Xinyu Miao and Zhonglong Yang**: methodology. **Hui Yang**: writing – review and editing. **Yunfei Guo**: writing – original draft, methodology, investigation, formal analysis, data curation.

## Funding

This work was supported by the National Key R&D Project (Grant 2024YFC2310300) and the Jiangsu Forestry Science and Technology Innovation and Promotion Project (Grant LYKJ[2024]16).

## Conflicts of Interest

The authors declare no conflicts of interest.

## Data Availability

The complete genome sequences (eight segments) of the isolates in this study have been deposited in the GISAID database under the accession number EPI_ISL_20324693. All other data supporting the findings of this study are available within the paper.

## References

[1] Zhou X. , Li Y. , and Wang Y. , et al.The Role of Live Poultry Movement and Live Bird Market Biosecurity in the Epidemiology of Influenza a (H7N9): A Cross-Sectional Observational Study in Four Eastern China Provinces, Journal of Infection. (2015) 71, no. 4, 470–479, 10.1016/j.jinf.2015.06.012. 26149187

[2] Wille M. and Holmes E. C. , The Ecology and Evolution of Influenza Viruses, Cold Spring Harbor Perspectives in Medicine. (2020) 16, no. 7, 10.1101/cshperspect.a038489. PMC7328453 31871237

[3] Garg S. , Reinhart K. , and Couture A. , et al.Highly Pathogenic Avian Influenza A(H5N1) Virus Infections in Humans, New England Journal of Medicine. (2025) 392, no. 9, 843–854, 10.1056/NEJMoa2414610. 39740051

[4] Nakhaie M. , Rukerd M. R. Z. , and Shahpar A. , et al.A Closer Look at the Avian Influenza Virus H7N9: A Calm Before the Storm?, Journal of Medical Virology. (2024) 96, no. 11, 10.1002/jmv.70090. 39601174

[5] Yang R. , Sun H. , and Gao F. , et al.Human Infection of Avian Influenza A H3N8 Virus and the Viral Origins: A Descriptive Study, The Lancet Microbe. (2022) 3, no. 11, e824–e834, 10.1016/S2666-5247(22)00192-6. 36115379

[6] Matrosovich M. , Tuzikov A. , and Bovin N. , et al.Early Alterations of the Receptor-Binding Properties of H1, H2, and H3 Avian Influenza Virus Hemagglutinins After Their Introduction Into Mammals, Journal of Virology. (2000) 74, no. 18, 8502–8512, 10.1128/JVI.74.18.8502-8512.2000. 10954551 PMC116362

[7] Gabriel G. , Herwig A. , and Klenk H. D. , Interaction of Polymerase Subunit PB2 and NP With Importin alpha1 is a Determinant of Host Range of Influenza a Virus, PLoS Pathogens. (2008) 4, 10.1371/journal.ppat.0040011. PMC2222953 18248089

[8] Ngai K. L. , Chan M. C. , and Chan P. K. , Replication and Transcription Activities of Ribonucleoprotein Complexes Reconstituted From Avian H5N1, H1N1pdm09 and H3N2 Influenza a Viruses, PLoS ONE. (2013) 8, 10.1371/journal.pone.0065038. PMC3672204 23750226

[9] Wan Z. , Gong J. , and Sang J. , et al.Mouse Adaptation of H6 Avian Influenza Viruses and Their Molecular Characteristics, Frontiers in Microbiology. (2022) 13, 10.3389/fmicb.2022.1049979, 1049979. 36466692 PMC9713515

[10] Li R. , Zhang T. , Xu J. , Chang J. , and Xu B. , Novel Reassortant Avian Influenza A(H3N8) Virus Isolated From a Wild Bird in Jiangxi, China, Microbiology Resource Announcements. (2019) 8, no. 46, 10.1128/mra.01163-19. PMC6856275 31727709

[11] Zhang T. , Li R. , Zhong P. , Chang J. , and Xu B. , Detection of Novel Reassortant H9N2 Avian Influenza Viruses in Wild Birds in Jiangxi Province, China, Veterinary Medicine and Science. (2021) 7, no. 3, 1042–1046, 10.1002/vms3.391. 33655656 PMC8136960

[12] Lee E. K. and Kang H. M. , Song, Surveillance of Avian Influenza Viruses in South Korea Between 2012 and 2014, Virology Journal. (2017) 14, 54. 28292308 10.1186/s12985-017-0711-y PMC5351195

[13] Altschul S. F. , Gish W. , Miller W. , Myers E. W. , and Lipman D. J. , Basic Local Alignment Search Tool, Journal of Molecular Biology. (1990) 215, no. 3, 403–410, 10.1016/S0022-2836(05)80360-2. 2231712

[14] Trifinopoulos J. , Nguyen L.-T. , von Haeseler A. , and Minh B. Q. , W-IQ-TREE: A Fast Online Phylogenetic Tool for Maximum Likelihood Analysis, Nucleic Acids Research. (2016) 44, no. W1, W232–W235, 10.1093/nar/gkw256. 27084950 PMC4987875

[15] Xiang C. Y. , Gao F. , and Jakovlić I. , et al.Using PhyloSuite for Molecular Phylogeny and Tree-Based Analyses, iMeta. (2023) 2, no. 1, 10.1002/imt2.87. PMC10989932 38868339

[16] Mei M. , Zhang X. , Wu Q. , Xu M. , and Zhao Y. , Virulence and Transmission Characteristic of H3N8 Avian Influenza Virus Circulating in Chicken in China, Virulence. (2026) 17, no. 1, 10.1080/21505594.2026.2613516, 2613516. 41504338 PMC12802998

[17] Kong X. , Guan L. , and Shi J. , et al.A Single-Amino-Acid Mutation at Position 225 in Hemagglutinin Attenuates H5N6 Influenza Virus in Mice, Emerging Microbes & Infections. (2021) 10, no. 1, 2052–2061, 10.1080/22221751.2021.1997340. 34686117 PMC8583753

[18] Gao R. , Gu M. , and Liu K. , et al.T160A Mutation-Induced Deglycosylation at site 158 in Hemagglutinin is a Critical Determinant of the Dual Receptor Binding Properties of Clade 2.3.4.4 H5NX Subtype Avian Influenza Viruses, Veterinary Microbiology. (2018) 217, 158–166, 10.1016/j.vetmic.2018.03.018. 29615249

[19] Mubareka S. , Lowen A. C. , Steel J. , Coates A. L. , García-Sastre A. , and Palese P. , Transmission of Influenza Virus via Aerosols and Fomites in the Guinea Pig Model, The Journal of Infectious Diseases. (2009) 199, no. 6, 858–865, 10.1086/597073. 19434931 PMC4180291

[20] Gao R. , Cao B. , and Hu Y. , et al.Human Infection With a Novel Avian-Origin Influenza a (H7N9) Virus, The New England Journal of Medicine. (2013) 368, 1888–1897, 10.1056/NEJMoa1304459. 23577628

[21] Fan S. , Deng G. , and Song J. , et al.Two Amino Acid Residues in the Matrix Protein M1 Contribute to the Virulence Difference of H5N1 Avian Influenza Viruses in Mice, Virology. (2009) 384, no. 1, 28–32, 10.1016/j.virol.2008.11.044. 19117585

[22] Kaplan B. S. , Kimble J. B. , and Chang J. , et al.Aerosol Transmission From Infected Swine to Ferrets of an H3N2 Virus Collected From an Agricultural Fair and Associated With Human Variant Infections, Journal of Virology. (2020) 94, 10.1128/jvi.01009-20. PMC7394887 32522849

[23] Yamayoshi S. , Yamada S. , and Fukuyama S. , et al.Virulence-Affecting Amino Acid Changes in the PA Protein of H7N9 Influenza A Viruses, Journal of Virology. (2014) 88, no. 6, 3127–3134, 10.1128/JVI.03155-13. 24371069 PMC3957961

[24] Yu R. , Jing X. , and Li W. , et al.Non-Structural Protein 1 From Avian Influenza Virus H9N2 is an Efficient RNA Silencing Suppressor With Characteristics that Differ From Those of Tomato Bushy Stunt Virus p19, Virus Genes. (2018) 54, no. 3, 368–375, 10.1007/s11262-018-1544-5. 29480423

[25] Chen H. , Bright R. A. , and Subbarao K. , et al.Polygenic Virulence Factors Involved in Pathogenesis of 1997 Hong Kong H5N1 Influenza Viruses in Mice, Virus Research. (2007) 128, no. 1-2, 159–163, 10.1016/j.virusres.2007.04.017. 17521765

[26] Katz J. M. , Lu X. , Tumpey T. M. , Smith C. B. , Shaw M. W. , and Subbarao K. , Molecular Correlates of Influenza a H5N1 Virus Pathogenesis in Mice, Journal of Virology. (2000) 74, no. 22, 10807–10810, 10.1128/JVI.74.22.10807-10810.2000. 11044127 PMC110957

[27] Yamada S. , Hatta M. , and Staker B. L. , et al.Biological and Structural Characterization of a Host-Adapting Amino Acid in Influenza Virus, PLoS Pathogens. (2010) 6, 10.1371/journal.ppat.1001034. PMC2916879 20700447

[28] Abdelwhab E. M. and Mettenleiter T. C. , Zoonotic Animal Influenza Virus and Potential Mixing Vessel Hosts, Viruses. (2023) 15, no. 4, 10.3390/v15040980, 980. 37112960 PMC10145017

[29] Olsen B. , Munster V. J. , Wallensten A. , Waldenström J. , Osterhaus A. D. , and Fouchier R. A. , Global Patterns of Influenza a Virus in Wild Birds, Science. (2006) 312, no. 5772, 384–388, 10.1126/science.1122438. 16627734

[30] Bao P. , Liu Y. , and Zhang X. , et al.Human Infection With a Reassortment Avian Influenza A H3N8 Virus: An Epidemiological Investigation Study, Nature Communications. (2022) 13, no. 1, 10.1038/s41467-022-34601-1, 6817. PMC9649012 36357398

[31] Liang J. , Li Q. , and Cai L. , et al.Adaptation of Two Wild Bird-Origin H3N8 Avian Influenza Viruses to Mammalian Hosts, Viruses. (2022) 14, no. 5, 10.3390/v14051097, 1097. 35632838 PMC9147613

[32] Solórzano A. , Foni E. , and Córdoba L. , et al.Cross-Species Infectivity of H3N8 Influenza Virus in an Experimental Infection in Swine, Journal of Virology. (2015) 89, no. 22, 11190–11202, 10.1128/JVI.01509-15. 26311894 PMC4645675

[33] Newton J. R. , Daly J. M. , Spencer L. , and Mumford J. A. , Description of the Outbreak of Equine Influenza (H3N8) in the United Kingdom in 2003, During Which Recently Vaccinated Horses in Newmarket Developed Respiratory Disease, Veterinary Record. (2006) 158, no. 6, 185–192, 10.1136/vr.158.6.185. 16474051

[34] Yang J. , Zhang Y. , and Yang L. , et al.Evolution of Avian Influenza Virus (H3) With Spillover Into Humans, China, Emerging Infectious Diseases. (2023) 29, no. 6, 1191–1201, 10.3201/eid2906.221786. 37069608 PMC10202863

[35] Sit T. H. C. , Sun W. , and Tse A. C. N. , et al.Novel Zoonotic Avian Influenza A(H3N8) Virus in Chicken, Hong Kong, China, Emerging Infectious Diseases. (2022) 28, no. 10, 2009–2015, 10.3201/eid2810.221067. 36037827 PMC9514342

[36] Suttie A. , Deng Y.-M. , Greenhill A. R. , Dussart P. , Horwood P. F. , and Karlsson E. A. , Inventory of Molecular Markers Affecting Biological Characteristics of Avian Influenza A Viruses, Virus Genes. (2019) 55, no. 6, 739–768, 10.1007/s11262-019-01700-z. 31428925 PMC6831541

[37] Skehel J. J. and Wiley D. C. , Receptor Binding and Membrane Fusion in Virus Entry: The Influenza Hemagglutinin, Annual Review of Biochemistry. (2000) 69, no. 1, 531–569, 10.1146/annurev.biochem.69.1.531. 10966468

[38] Lin J. , Li Y. , and Luo H. , et al.Epidemiological Investigation and Characterization of Avian Influenza A H3N8 Virus in Guangdong Province, China, Animals. (2025) 15, 10.3390/ani15233377. PMC12691397 41375434

[39] Zhou S. , Zhang Y. , and Liu S. , et al.Pathogenicity of Novel H3 Avian Influenza Viruses in Chicken and Development of a Promising Vaccine, Viruses. (2025) 17, no. 3, 10.3390/v17030288, 288. 40143220 PMC11946779

[40] Mao Q. , Zhou S. , and Liu S. , et al.Emergence of Novel Reassortant H3N3 Avian Influenza Viruses With Increased Pathogenicity in Chicken in 2023, Emerging Microbes & Infections. (2024) 13, no. 1, 10.1080/22221751.2023.2287683, 2287683. 37990831 PMC10795584

[41] Germeraad E. A. , Sanders P. , Hagenaars T. J. , de Jong M. C. M. , Beerens N. , and Gonzales J. L. , Virus Shedding of Avian Influenza in Poultry: A Systematic Review and Meta-Analysis, Viruses. (2019) 11, no. 9, 10.3390/v11090812, 812. 31480744 PMC6784017

[42] Wang P. , Fu J. , and Wu H. , et al.Isolation and Characterization of a Novel Reassortant H3N8 Avian Influenza Virus From Chicken in Eastern China, Virus Genes. (2026) 62, no. 1, 106–115, 10.1007/s11262-025-02200-z. 41324788

